# Genetic Factors of Autoimmune Diseases

**DOI:** 10.1155/2016/3476023

**Published:** 2016-01-26

**Authors:** Fulvia Ceccarelli, Nancy Agmon-Levin, Carlo Perricone

**Affiliations:** ^1^Lupus Clinic, Rheumatology, Department of Internal Medicine, Sapienza University of Rome, Viale del Policlinico 155, 00161 Rome, Italy; ^2^The Zabludowicz Center for Autoimmune Diseases, Sheba Medical Center, The Sackler Faculty of Medicine, Tel-Aviv University, 5265601 Tel Hashomer, Israel

The multifactorial pathogenesis of autoimmune disease has been widely confirmed; indeed, several evidences underline the interaction between genetic and environmental factors in determining the development of autoimmunity [1]. The higher concordance ratio between monozygotic twins compared to dizygotic twins or other siblings confirmed the role of genetic factors in the pathogenesis of many autoimmune diseases. More recently, genome wide association studies have allowed the identification of several genetic loci associated not only with disease susceptibility, but also with specific clinical manifestations or outcomes [1]. The proteins encoded by genes associated with autoimmune diseases are involved in several inflammatory mechanisms, such as antigen presentation, type I interferon, Toll-like receptor and NF-*κ*B signaling, B-cell and T-cell function, apoptosis, and clearance of cellular debris and immune complexes [2]. Genetic variants could induce proteins modifications, in terms of production rate and function, with possible changes in the related processes. Moreover, different autoimmune diseases are linked with the same genetic modifications, suggesting a shared genetic pathway to loss of tolerance and induction of autoimmunity [1, 2].

The present special issue includes 2 reviews and 12 research articles, focusing on aspects related to genetic factors in determining autoimmune diseases susceptibility and phenotype. Interestingly, in addition to classical autoimmune diseases, such as Systemic Lupus Erythematosus (SLE) and Sjögren's Syndrome (SjS), others have been investigated, making this special issue even more intriguing. Looking at the broad spectrum of genetics and autoimmunity, the role of HLA-DRB1 alleles has been evaluated in a large cohort of patients affected by different autoimmune diseases, identifying associations between specific alleles and different diseases and the HLA-DRB13 underrepresentation in all diseases evaluated [e.g., SLE, Psoriasis (PS), Psoriatic Arthritis (PsA), Rheumatoid Arthritis (RA), Systemic Sclerosis (SSc), Multiple Sclerosis (MS), and Myasthenia Gravis (MG)]. A very recent paper has confirmed such role of the HLA-DRB1^*∗*^13 showing that some alleles are associated with protection from ACPA-positive RA, but not with significant protection from ACPA in individuals without RA. These data indicate that HLA-DRB1^*∗*^13 mainly affects the onset of ACPA-positive RA in ACPA positive non-RA individuals [3].

Moreover, the special issue includes an interesting evaluation of familial aggregation of first-degree relatives as well as segregation analysis in families presenting with autoimmune diseases. Polyautoimmunity and multiple autoimmune syndrome seem to be dependent traits, while gender, age, and age of onset are interrelated factors that also influence autoimmunity.

Five studies addressed the role of genetic factors in SLE susceptibility and phenotypes. In particular, the association between the polymorphisms of the gamma-aminobutyric acid receptor subunit pi (GABRP) gene, neurological diseases, and SLE susceptibility has been investigated, revealing significant differences in terms of genotype frequencies (rs929763, rs732157, and rs3805455) among SLE patients compared with the control group. The first evidence for the role of cyclic AMP-responsive element modulator *α* (CREM*α*) polymorphisms in SLE susceptibility has been suggested in this issue. CREM proteins are members of the leucine zipper protein superfamily of nuclear transcription factors and act as regulators of cAMP-mediated signal transduction. Moving from the evidence demonstrating CREM*α* overexpression in T cells from SLE patients, specific CREM*α* SNPs (rs2295415, rs1057108) seem to be associated with SLE susceptibility.

Genetic factors are associated not only with SLE susceptibility, but also with specific disease phenotypes: data published so far deriving from the analysis of small cohorts, which did not enable conclusive results. Remarkably, the majority of studies published so far on this linkage focused on the influence of genetic factors in determining renal manifestations. In this special issue, a relationship between ATG5 SNPs and lupus nephritis has been suggested, by using new systemic genetics approach.

Moreover, the expansion of CD25^high^  FoxP3^high^ B regulatory cells in SLE patients is an intriguing topic. This cell subset, characterized by high expression of IL-10, was found to be increased in SLE patients and in correlation with disease activity. This result suggests that these cells expansion could represent the attempt of the regulatory immune responses to maintain self-tolerance and to suppress SLE disease activity. Other systemic and organ specific autoimmune diseases have also been linked to genetic factors. In SjS and SjS-related lymphomagenesis, Sjögren's disease was linked with genetic variants in the major histocompatibility complex (MHC) locus. Moreover, genetic variants outside the MHC locus, such as those involving genes of the type I interferon pathway, NF-*κ*B signaling, B-cell and T-cell function, and methylation processes, have also been described.

Two further studies evaluated pediatric autoimmune disease, such as juvenile idiopathic arthritis (JIA) and autoimmune hepatitis (AIH). An association between poor prognosis in JIA patients and the TRAF1/C5 gene locus was suggested, although larger studies are required to confirm this result. Furthermore, the role of IL-13, IL-4RA, and HLA-DRB1 polymorphisms in AIH type I has been evaluated, identifying an association with specific genetic variants.

In another liver disease, Primary Sclerosing Cholangitis (PSC), the expression of sulfotransferase 2A1 (SULT2A1) enzyme has been estimated, founding a modification of SULT2A1 expression, probably related to the impaired hepatoprotection. Moreover, miRNA analysis suggested a role for miR-378a-5p in the SULT2A1 expression.

A large number of SNPs in genes previously associated with skin autoimmune disease plaque psoriasis (PS) have been investigated in a large population. The distinction between type I (early-onset, <40 years) and type II (late-onset, ≥40 years) PS reveals an association between early-onset and polymorphisms of the CLMN, FBXL19, CCL4L, C17orf51, TYK2, IL-13, SLC22A4, CDKAL1, and HLA-B/MICA genes. Moreover, a significant association between age at onset and gene variants of PSORS6, TNF-*α*, FCGR2A, TNFR1, CD226, HLA-C, TNFAIP3, and CCHCR1 was also identified. These data suggested the role of genetic factors in determining the age of onset in patients with plaque psoriasis.

Another organ specific autoimmune disease, uveitis, has been investigated for the interaction between disease and IL-6 gene polymorphism and HLA-B27. A significantly higher frequency of the minor allele for rs1800795 in patients with intermediate uveitis compared to controls has been identified, suggesting a role of IL-6 as therapeutic target in patients with HLA-B27 associated uveitis. Last but not least, a study on 235 hemochromatosis probands was performed, demonstrating the presence of autoimmune conditions in high percentage of subjects, with Hashimoto's thyroiditis being most prevalent. Notably, the risk of autoimmunity in this scenario was not associated with any HLA haplotype.

In conclusion, the present special issue adds interesting data concerning genetic factors associated with different systemic and organ specific autoimmune diseases, evaluating the genetic impact both on disease susceptibility and on disease phenotypes. Conspicuously, there is an urgent need for more studies utilizing large cohorts that will broaden our knowledge as currently it is estimated that only about 15% of the genetic factors contributing to autoimmune disease susceptibility have been identified [4]. Moreover, besides genetic association studies, functional analyses aiming at unveiling the mechanistic role of each factor should be performed. Novel techniques, including next-generation sequencing studies, will further contribute and expand our understanding of the genetic basis of autoimmunity.

*Fulvia Ceccarelli* *Fulvia Ceccarelli* *Nancy Agmon-Levin* *Nancy Agmon-Levin* *Carlo Perricone* *Carlo Perricone*
